# THE ROLE OF TIME PREFERENCES IN THE INTERGENERATIONAL TRANSFER OF SMOKING

**DOI:** 10.1002/hec.2987

**Published:** 2013-08-19

**Authors:** Heather Brown, Marjon van der Pol

**Affiliations:** a Institute of Health and Society, Newcastle UniversityNewcastle upon Tyne, UK; b Health Economics Research Unit, University of AberdeenAberdeen, UK

**Keywords:** intergenerational transfer, smoking, time preference, decomposition analysis

## Abstract

Evidence suggests that maternal and offspring smoking behaviour is correlated. Little is known about the mechanisms through which this intergenerational transfer occurs. This paper explores the role of time preferences. Although time preference is likely to be heritable and correlated with health investments, its role in the intergenerational transmission of smoking has not been explored previously. This is the first paper to empirically test this. Data (2002, 2003, 2004, 2006 and 2008) from the Household, Income and Labour Dynamics in Australia are used. Estimates by using a pooled probit model show that there is not a direct effect of maternal time preference, measured in terms of financial planning horizon, on the likelihood that their offspring is a smoker. However, there is an indirect effect of maternal time preference. Sons of mothers that are smokers and have a shorter planning horizon are 6% more likely to smoke than if their mother had a longer planning horizon, and daughters of mothers that smoke with a shorter planning horizon are 7% more likely to smoke themselves than if their mother had a longer planning horizon.

## 1. INTRODUCTION

The correlation between parents' and their offspring's smoking behaviour is well established (for example Wickrama *et al*., 1999; Shenassa *et al*., 2003). However, the mechanisms behind the intergenerational transmission of smoking are still unclear. To inform interventions and policy aimed at reducing smoking, it is crucial to gain a better understanding of the mechanisms contributing to persistence across generations. Both shared genetics and shared environment between parents, and their offspring are likely to play a role. This paper focuses on the role of time preference. Bowles and Gintis (2002) proposed that the intergenerational transmission of attitudes and personality traits such as time preference play an important role in the intergenerational correlation of economic outcomes. Delaney *et al*. (2011) found that about 5% of the correlation in offspring grades and parental education can be attributed to time preference.

Time preference can also be hypothesised to play a role in the intergeneration transmission of smoking as there is an established theoretical and empirical relationship between time preference and health investments such as smoking behaviour. In the canonical model of the demand for health, the Grossman model, health behaviours are modelled as investments in human capital (Grossman, 1972). Choices regarding the level of health investments are made so that the expected discounted utility over a lifetime is maximised. Health investments such as smoking cessation typically involve short-term costs and long-term benefits. A higher discount rate will reduce the value of future health and therefore reduce the level of health investment. Most studies have found a significant association between high time preference and smoking (for example Scharff and Viscusi, 2011).

Little is known about how time preferences are formed, but it can be hypothesised that parents transmit preferences including time preferences to their offspring. Time preference may be ‘learned’ through imitation where parents act as role models to their offspring. Parents may also invest resources to change their children's preferences (Bisin and Verdier, 2001). The Becker and Mulligan (1997) model specifically considers how parents influence their children's rate of time preference by investing resources to make their children more future oriented. Limited empirical evidence suggests that parents' and their offspring time preference are correlated (Knowles and Postlewaite, 2004; Webley and Nyhus, 2006; Reynolds *et al*., 2009).

Although time preference is likely to be heritable and correlated with health investments, its role in the intergenerational transmission of smoking has not been explored previously. This is the first paper to empirically test this. The role of time preference is investigated after controlling for other potential pathways including education and employment status. The paper also explores any gender asymmetries. Previous literature has shown differences across genders in the intergenerational transmission of health behaviours. For example, Wickrama *et al*. (1999) found a significant correlation between mother's and daughter's health behaviours and father's and son's health behaviours but no significant cross-gender correlations. This paper explores whether the role of time preference in the intergenerational transmission of smoking differs across gender lines.

## 2. DATA

Data from the Household Income Labour Dynamics of Australia (HILDA) are used. The HILDA is a nationally representative annual household-based panel survey, which began in 2001 (Summerfield *et al*., 2011). The panel members are followed over time, and each household member over the age of 15 years is interviewed.

The sample is restricted to young adults between the ages of 16 and 25 years and their mothers (our entire sample still resides with their mothers). The analysis focuses on the intergenerational transmission from mothers to their offspring because of the traditional role of mothers as primary caregivers. An unbalanced panel is used, and sample members need to be present in at least one wave of data. Waves from 2002, 2003, 2004, 2006 and 2008 include information on smoking and time preference and are utilised in the analysis. There are 3167 young adults and 1901 mothers in the sample.^1^

### 2.1. Smoking

Smoking status is a binary variable that equals one if respondents report any level of tobacco consumption and is equal to zero otherwise. Further information on the variable can be found in APPENDIX A. Offspring smoking status is modelled as a function of maternal smoking status.

### 2.2. Time preferences

Time preferences are not readily observed, and empirical studies have to rely on proxies. Commonly used proxies relate to financial savings behaviour. Financial planning horizon has been used in several studies as a proxy for time preference (for example Samwick, 1998; Picone *et al*., 2004; Khwaja *et al*., 2007). Adams and Nettle (2009) showed that planning horizon and time preference rate, measured by using hypothetical trade-offs over time, are correlated. There are other factors that are likely to be associated with planning horizon such as socioeconomic status and life expectancy, and these are controlled for in the analysis.

The current study also uses a proxy for financial planning horizon.^2^ A binary variable was created that equals one if respondents report planning horizons longer than a year and zero otherwise.

The raw correlation in maternal and offspring time preference is 0.13, *p* < 0.001. The analysis will investigate how maternal time preference contributes to the intergenerational transmission of smoking.

### 2.3. Other mediating variables

Employment status, as well as if the young adult is a full-time student, are included in all models.

### 2.4. Household and offspring characteristics

Household characteristics such as two dummies for local area deprivation calculated from an index of relative socioeconomic disadvantage,^3^ and the log of equivalised household income are included in all models. Maternal socioeconomic characteristics such as employment status, maternal marital status and educational attainment are included in the analysis. Maternal time preference is also included in all model specifications. Young adult individual characteristics such as age and age squared are also included in the analysis.

The descriptive statistics for the sample are presented in APPENDIX C.

## 3. ECONOMETRIC FRAMEWORK

We start by estimating a pooled probit model for the determinants of young adult smoking:(1)
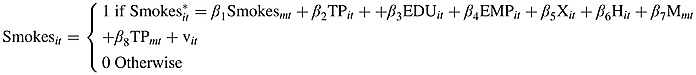


The *i* subscript denotes young adults, the *m* subscript denotes mothers, the *f* subscript denotes family, and *t* represents time. Smokes*_it_* is a binary variable for young adult (*i*) in period *t*. Individual (*i*) is a smoker in period *t* if 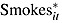 = 1. The scalar Smokes*_mt_* contains a dummy variable for the mother's smoking status; the scalar TP*_**i**t_* contains a dummy variable for the young adult's planning horizon, proxy for time preference; the scalar EDU*_it_* contains a dummy variable for the young adult's full-time student status; the vector EMP*_it_* contains dummy variables for the young adult's employment status; the vector X***_it_*** contains variables related to age; the vector H*_ft_* contains variables related to household characteristics; the vector M*_mt_* contains variables related to maternal socioeconomic characteristics; and the scalar TP***_mt_*** contains a variable for maternal time preference proxy. The associated coefficient of parameters to be estimated for the scalars are *β*_1_, *β*_2,_*β*_3_, *β*_8_ and for the vectors are *β*_4,_*β*_5_, *β*_6_, *β*_7_. v_*it*_ is a random error term. The standard errors are clustered by the mother's personal identification number to account for multiple children by a single mother.

To investigate if maternal time preference has an indirect effect (i.e. is a contributing factor) to the intergenerational transmission of smoking, we introduce an interaction term for maternal smoking and time preference *β*_9_Smokes_*mt*_ * TP_*mt*_ to Equation (1). If time preferences are indirectly transmitted from mothers to their offspring, we would expect the intergenerational transmission of smoking to be stronger for mothers who have a shorter planning horizon (higher time preferences). Marginal effects calculated from interaction terms estimated from nonlinear models do not have a straightforward interpretation (Ai and Norton, 2003; Norton *et al*., 2004; Cornelißen and Sonderhof, 2009). To provide a meaningful interpretation of our results, following Buis (2010), we calculate multiplicative effects. The interaction effect then tells us how much the effect of maternal smoking differs by the mother's planning horizon.

The robustness of the results is examined by reestimating the models using alternative classifications of the smoking and time preference variables. We estimate models with categorical classification and alternative thresholds for the binary classification of both variables.

## 4. RESULTS

The first step of the estimation strategy is to determine the factors influencing young adult smoking by estimating Equation (1). Results are shown in Table 1. Marginal effects are estimated. The results confirm that maternal smoking significantly increases the likelihood of smoking. The results also confirm that young adults with a lower time preference rate (longer planning horizon) are less likely to smoke. Maternal time preference is not significant for either gender suggesting that there is not a direct effect of maternal time preference on the likelihood of smoking.

**I:** Determinants of smoking

	Men		Women	
Smoking	ME	SE	ME	SE
Longer planning horizon	−0.050**	(0.021)	−0.054***	(0.018)
Age	0.115**	(0.056)	−0.008	(0.057)
Age squared	−0.003*	(0.001)	0.000	(0.001)
Employed	−0.005	(0.027)	0.007	(0.024)
Unemployed	0.066*	(0.037)	0.111**	(0.045)
Full-time student	−0.132***	(0.024)	−0.144***	(0.025)
Mother (smoker)	0.187***	(0.036)	0.152***	(0.037)
Mother (high school)	−0.009	(0.039)	−0.024	(0.034)
Mother (some college)	0.003	(0.031)	0.020	(0.032)
Mother (university)	0.036	(0.038)	−0.004	(0.036)
Most deprived deciles (1–3)	−0.006	(0.033)	0.031	(0.030)
Deciles of deprivation (4–7)	−0.004	(0.033)	−0.023	(0.031)
Log household income	−0.023	(0.023)	0.002	(0.021)
Mother (employed)	−0.013	(0.029)	−0.033	(0.029)
Mother (unemployed)	−0.019	(0.074)	0.019	(0.058)
Mother (married)	0.018	(0.059)	−0.078	(0.053)
Mother (divorced)	0.057	(0.071)	0.015	(0.052)
Mother (longer planning horizon)	−0.006	(0.021)	−0.002	(0.021)
Observations	2143		1917	

Standard errors in parentheses (columns labelled SE) and the columns labelled ME show marginal effects.

* *p* < 0.1. It is estimated by using a probit model where young adult smoking is the dependent variable. Standard errors are clustered by mother's identification number to control for multiple family observations;

** *p* < 0.05;

*** *p* < 0.01.

In the male models, there is a significant relationship between age and smoking. For both men and women, being unemployed significantly increases the likelihood of smoking. For both genders, being a full-time student significantly decreases the likelihood of smoking. The significance of the proxy for time preference (longer planning horizon) and full-time education suggests that the longer planning horizon is capturing another dimension of future orientation beyond that of foregone earnings by choosing to remain in education. None of the household characteristics are significant. For women, having a mother that is married has a marginally significant negative effect on the likelihood of smoking.

Table 2 shows the interaction of maternal time preference and smoking to determine if there is an indirect effect of maternal time preference on the intergenerational transmission of smoking. We calculate the multiplicative effects of maternal time preference on the likelihood of smoking by subtracting the difference in the likelihood of smoking for offspring of mother's that smoke with a longer and shorter planning horizon. Men that have a mother who is a smoker and has a shorter planning horizon are 6% more likely to smoke compared with those who have a mother who is a smoker and has a longer planning horizon. Women that have a mother that smokes and a shorter planning horizon are 7% more likely to smoke those who have a mother is a smoker and has a longer planning horizon. Maternal time preference works indirectly through smoking status, but there is not a direct effect of maternal time preference as was shown in Table 1. Time preference does not mediate the likelihood of smoking if one's mother does not smoke.

**Table II:** Interaction effects of maternal time preference and smoking

	Men		Women	
Mother short planning horizon and nonsmoker	0.19***	(0.02)	0.16***	(0.02)
Mother short planning horizon and smoker	0.41***	(0.05)	0.34***	(0.04)
Mother longer planning horizon and nonsmoker	0.20***	(0.02)	0.17***	(0.02)
Mother longer planning horizon and smoker	0.35***	(0.05)	0.28***	(0.05)

Standard errors in parentheses.

* *p* < 0.10. Marginal effects are shown. Interaction effects were estimated by using Equation (1) and the stata version 12, (StataCorp, College Station, Texas, USA) post-estimation command margins. Baseline effects are also shown to estimate the multiplicative effects of time preference on the likelihood of smoking for offspring of mother's that are smokers;

** *p* < 0.05;

*** *p* < 0.01.

We run a number of robustness checks on the classification of the smoking and time preference variable. We estimate ordered probit models using a categorical classification of smoking; some specifications include a categorical variable for time preference. We also estimate probit models using two alternative thresholds for smoking classification: (i) restricting the base category of nonsmokers to never smokers only; and (ii) classifying infrequent smokers in the nonsmokers category. In some probit specifications of the determinants of smoking, a categorical time preference variable is included, and in other models, an alternative binary classification of time preference for young adults and mothers where longer planning horizon is restricted to those who claim they plan their finances more than 2 years in advance is estimated. Finally, we estimate some pooled probit model specifications using alternative binary thresholds for both smoking and time preference.

Results from the robustness checks are similar to those found in Tables1 and 2. Longer time preference is negatively and significantly associated with the likelihood of smoking. Maternal smoking has a positive and significant effect on the likelihood of smoking, and maternal time preference is not significant in any specification. An indirect effect of maternal time preference on the intergenerational transmission of smoking is present under all alternative specifications.

## 5. DISCUSSION

This paper investigated the role of time preference in the transmission of smoking from mothers to their offspring. The results show that the maternal time preference measured by financial planning horizon has an indirect effect in explaining the correlation in maternal and offspring smoking behaviour but not a direct effect. The indirect effect of time preference is slightly larger for women suggesting that the role of time preferences is more important along gender lines. We hypothesise that this indirect effect is, at least partly, the result of the transmission of time preferences, which were shown to be correlated between mothers and their offspring.

The results rely on the assumption that planning horizon is a good proxy for time preference. The proxy may not only measure time preference but also capture other unobserved characteristics such as the availability of household resources. However, planning horizon was shown to be correlated with smoking behaviour even when controlling for both maternal and offspring socioeconomic status.

The sample in this study all resided with their mothers; thus, the results may not be generalisable to the population level. Information from the Australian Bureau of Statistics from 2006 found that approximately 42.7% of individuals in the 20–24 years age group lived with their parents. However, it was also found that 46% of young Australians who moved out of the family home moved back within 3 years.

It should be noted that the correlations in smoking behaviour and time preference may not necessarily reflect causal relationships (Bowles and Gintis, 2002). However, the findings provide some indication that time preference plays a role in the intergenerational transmission of smoking. It suggests that those concerned with understanding and developing interventions and policies to reduce smoking should consider the transmission of preferences.

## Figures and Tables

**Figure 1:**
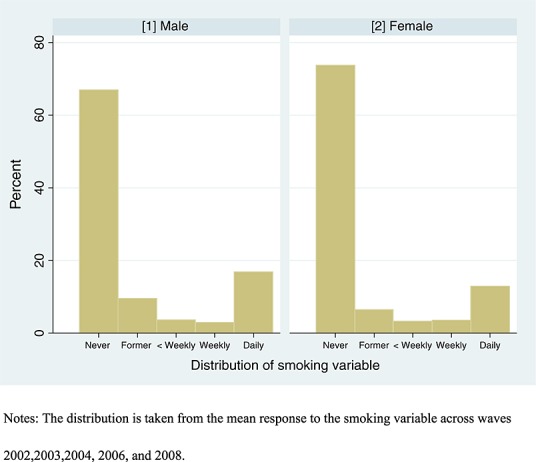
Distribution of the smoking variable for young adults separated by gender

**Figure 2:**
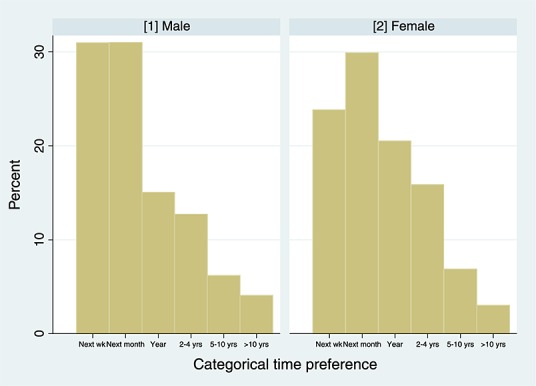
Distribution of time preference variable for young adults separated by gender

**Figure 3:**
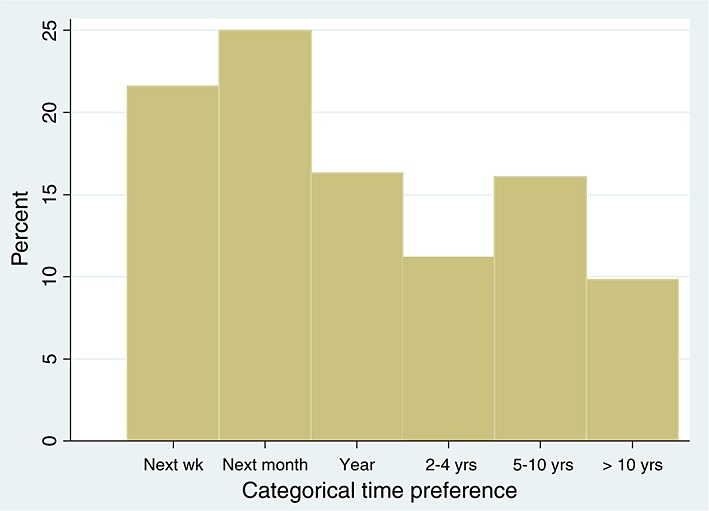
Distribution of time preference for mothers

## Footnotes

1 The numbers are not equal because 47% of the mothers in the sample have between two and four children in the 16–25 years age group.

2 Further details on the financial planning variable can be found in APPENDIX B.

3 Index of socioeconomic disadvantage is calculated by using areas defined from the 2001 census and is based on attributes such as low income, low educational attainment and high unemployment. There are 10 deciles with higher numbers indicating lower levels of deprivation.

## APPENDIX A THE SMOKING VARIABLE

The smoking variable is asked to respondents in the self-completion questionnaire: ‘Do you smoke cigarettes or any other tobacco product?’

The respondent has the option of choosing: (i) nonsmoker; (ii) former smoker; (iii) daily smoker; (iv) weekly smoker (not daily); and (v) smoke less frequently than weekly.

The distribution of the smoking variable for young adults is shown in Figure 1.

The distribution is taken from the mean response to the smoking variable across waves 2002, 2003, 2004, 2006 and 2008.

## APPENDIX B FINANCIAL PLANNING QUESTION FROM THE HILDA SURVEY

The financial planning horizon question is asked to respondents in the self-completion questionnaire: ‘In planning your savings and spending which of the following time periods is most important to you?’

The respondent has the option of choosing: (i) next week; (ii) next few months; (iii) next year; (iv) next 2–4 years; (v) next 5–10 years; and (vi) more than 10 years ahead.

The distribution of the time preference variables are shown in Figures2 and 3.

## Appendix

**Table 1:** APPENDIX C DESCRIPTIVE STATISTICS

	Men	Women
Smokes	0.20 (0.40)	0.16 (0.37)
Individual Characteristics		
Age	18.88 (2.54)	18.64 (2.43)
Employed	0.67 (0.47)	0.69 (0.46)
Unemployed	0.10 (0.30)	0.07 (0.25)
Full-time student	0.50 (0.50)	0.58 (0.49)
Longer planning horizon	0.37 (0.48)	0.45 (0.50)
Household Characteristics		
Deciles of deprivation (4–7)	0.25 (0.43)	0.27 (0.44)
Most deprived deciles (1–3)	0.48 (0.50)	0.50 (0.50)
Log household income	10.00 (0.62)	10.04 (0.62)
Maternal Characteristics		
Smoker	0.17 (0.38)	0.16 (0.36)
Employed	0.74 (0.44)	0.77 (0.42)
Unemployed	0.02 (0.16)	0.03 (0.16)
Married	0.81 (0.40)	0.79 (0.41)
Divorced	0.15 (0.36)	0.16 (0.37)
High school	0.13 (0.34)	0.14 (0.35)
Post-high school	0.28 (0.45)	0.26 (0.44)
University	0.23 (0.42)	0.24 (0.43)
Longer planning horizon	0.52 (0.49)	0.55 (0.50)
*n*	4213	3814

All variables are the means across waves 2002, 2003, 2004, 2006 and 2008. Age is measured in years, the log of household income is measured in Australian dollars, and all other variables are measured in percentages.
